# Notes and News

**Published:** 1899-03-18

**Authors:** 


### Notes and News.

(Collected and Communicated.)

### HOSPITAL MEETINGS.

West End Hospital, 73, Welbeck Street, W.—Annual General Meeting at four p.m., on Thursday, March 23rd, at the Hospital.

Hospital and Home for Incurable Children, 2, Maida Vale, W.—Annual Meeting at three p.m., on Saturday, March 18th.

British Lying-in Hospital, Endell Street, W.C.—Annual General Meeting at five p.m., on Tuesday, 21st inst., at the Hospital.

East End Mothers' Home, 394 and 396, Commercial Road, E.—Annual Meeting at half-past three p.m., on Friday, 24th inst., at the Home. (Corrected notice.)

East London Nursing Society.—Annual Meeting at three p.m., on Thursday, 23rd inst., at St. Martin's Church Vestry Hall, Charing Cross.

In connection with the opening of the new building of the Middlesex Hospital Medical School, Dr. Frederick Hetley, a former student, has contributed the sum of £1,000 to perpetuate the Hetley Clinical Prize of £25 per annum founded in 1884.

The Royal Agricultural Society has just issued a leaflet on "Tuberculosis in Dairy Stock," which should be of material service to dairy farmers. The pamphlet gives information concerning the separation and general treatment of infected stock, and on the best methods for carrying out a system of prevention. Copies may be had free on application to the Secretary of the Society, 13, Hanover Square, W.

Mr. Chamberlain's announcement of the establishment of a school for the study of tropical diseases has been received with very general approbation. In his speech on the subject last week Mr. Chamberlain said that during his tenure of the Colonial Office nothing had given him more pain and anxiety than the mortality amongst young and promising officers from these diseases, mortality which he believed to be preventable. The steps now being taken to ensure that medical men and nurses going out to work in the colonies shall first have special clinical and practical instruction in the treatment of the particular diseases with which they are likely to be brought in contact, will go far towards remedying this state of things. The school is to be in connection with the "Dreadnought" Hospital at Greenwich, and the necessary funds will be provided partly by Government grant, partly by private subscription, and partly by contributions from the colonies.

A new ward was opened at the London Temperance Hospital on Thursday, March 16th. The opening ceremony took place immediately before the annual public meeting, presided over by the Duke of Westminster, K.G.

H.R.H. the Duke of York has consented to become president of the Royal Humane Society, in succession to the Duke of Argyll, who retires after 40 years' office. The Prince of Wales has expressed his willingness to become a vice-patron of the society.

It is satisfactory to note that Lord Wemyss has given notice of his intention to ask the House of Lords, on an early date, to agree to a resolution for the appointment of a Royal Commission to inquire into the administration and working of the Poor Law. The grievous anomalies of the present Poor Law, unsuited as it is in so many ways to modern conditions of life, are beginning to rouse much public comment, and Lord Wemyss will be doing good service if he carries his resolution.

We mention elsewhere that the English losses by disease during the Crimean War were about ten times as great as those inflicted by the enemy; but the official return from the office of the American Adjutant-General discloses a still greater disproportion between the two sources of mortality, and emphasises the fact that, especially if war be made in a hurry and without adequate medical preparation and foresight, a similar disproportion must always be expected to obtain. Between May 1st, 1898, and February 18th in the present year, 329 men of the American troops were killed in action, and 125 died of their wounds, a total of 454; while 5,277 died from disease, either in the United States, in Cuba, in Puerto Rico, or in the Philippines.

Codrington College, Barbadoes, the chief University of the West Indies, is in imminent danger of financial collapse, and an appeal is being issued by the West India Committee (Billiter Square Buildings, London, E.C.), signed by the Archbishop of Canterbury and other friends and sympathisers, for subscriptions to avert its closure. The estates left by its founder, General Codrington, owing to the present depression in the sugar trade, are no longer sufficient to provide for its maintenance, and it rests with the public to see that this important "Christianising and civilising" centre shall not be allowed to cease its good work. A minimum emergency fund of £5,000 is required by May 1st.

In consequence of the fatal results of introducing the plague bacilli into the laboratory in Vienna, a medical man in Melbourne has been obliged to hand over his specimens to the Government for destruction. Dr. Haydon, the medical man in question, had been sent to India from England on a commission respecting the plague, and was staying in Melbourne with specimens of bacilli in his possession. He demanded £300 as compensation for the surrender of his property, but the Government evaded the demand by confiscating the specimens on the plea that the duty on the gelatine in which they were kept had not been paid as it should have been. Dr. Haydon stated he retained the cultures for the purpose of inoculation against the disease should it be introduced into the colony, and was very indignant at the treatment received from the authorities.

